# Genome sequencing analysis of a novel thermophilic strain *Geobacillus* sp. CX412

**DOI:** 10.3389/fmicb.2022.1035311

**Published:** 2022-11-11

**Authors:** Xin Li, Wei Zhang, Xin-Ru Zhong, Hao-Xuan Han, Bin Dong

**Affiliations:** ^1^School of Environmental Science and Engineering, Tongji University, Shanghai, China; ^2^School of Environment and Architecture, University of Shanghai for Science and Technology, Shanghai, China; ^3^YANGTZE Eco-Environment Engineering Research Center, China Three Gorges Corporation, Beijing, China

**Keywords:** *Geobacillus* sp. CX412, thermophile, transposons, β-lactam resistance, lignocellulose

## Abstract

The thermophilic spore-forming strain *Geobacillus* sp. CX412 was isolated from hot spring soil in Tengchong City, Yunnan Province, China. We sequenced the complete genome of *Geobacillus* sp. CX412 using PacBio SMRT Sequencing. Genome-scale phylogenetic analysis and average nucleotide identity (ANI) results indicated that *Geobacillus* sp. CX412 is a novel species in the genus *Geobacillus*. The metabolic potential of *Geobacillus* sp. CX412 based on COG, KEGG, and CAZymes analysis demonstrated that *Geobacillus* sp. CX412 was a highly adaptable strain with an unusually high number of 73 annotated transposons in the genome, which is relatively rare in *Geobacillus*. Compared with the near-derived strains, it was found that *Geobacillus* sp. CX412 has the unique β-lactam resistance and more active metabolism (more than 50.5–100.1%). Additionally, its genome encodes glycoside hydrolases and other genes related to lignocellulose breakdown, suggesting that *Geobacillus* sp. CX412 has a considerable biomass degradation potential. Thus, *Geobacillus* sp. CX412 is a new thermophilic bacterial species that add to the increasing repertoire of known lignocellulose degraders.

## Introduction

*Geobacillus* were categorized initially as “Group 5” in the genus *Bacillus*. They were subsequently split into the new genus based on 16S rRNA gene sequence analysis, phenotypic characterization, and DNA-DNA hybridization experiments, including thermophilic gram-positive spore-forming bacteria that form phylogenetically consistent clades within the *Bacillus* family (Nazina et al., 2001; Chen et al., 2015; Brumm et al., 2016). In 2016, the genus *Geobacillus* was subdivided into two genera based on whole-genome approaches, with the addition of *Parageobacillus* (Aliyu et al., 2016, 2018; Najar et al., 2020). In 2020, Reclassification of *Geobacillus galactosidasius* and *Geobacillus yumthangensis* as *Parageobacillus galactosidasius* comb. nov. and *Parageobacillus yumthangensis* comb. nov., respectively (Najar et al., 2020). Therefore, *Geobacillus* and *Parageobacillus* are relatively similar in the phylogenetic tree and often cross over.

*Geobacillus* species have been found mainly in hot springs in the United States (Brumm et al., 2015a,b), Africa (Hawumba et al., 2002), and Russia (Nazina et al., 2004), the Mariana Trench (Takami et al., 2004), deep-sea vents (Maugeri et al., 2002), high-temperature oilfields (Kuisiene et al., 2004), a corroded pipeline in an extremely deep well (Popova et al., 2002), and composting materials (Bhalla et al., 2013; Li et al., 2014; Brumm et al., 2016). It demonstrates the ability of *Geobacillus* to thrive in this diverse and often harsh environment and suggests that these species have enzymes suitable for application in challenging industrial environments (such as enzymes that efficiently break down lignocellulose) (Bouzas et al., 2006; Bergquist et al., 2014; Chen et al., 2015). *Geobacillus* species can grow in high-temperature environments (up to 70°C or more), and the advantages of using thermophilic bacteria as whole-cell biocatalysts include reduced risk of contamination and accelerated biochemical processes in fermentation (Chen et al., 2015). Composting Materials, as the main sources of thermal bacteria, also imply that thermal bacteria would use organic matter to self-reproduce during composting. When antibiotic production residue is used as compost substrate, due to the inhibitory and poisoning effect of antibiotics, thermal bacteria may not be able to reproduce and grow well, which would reduce the composting effect (Yang et al., 2016). Therefore, finding bacteria that may resist antibiotics under high-temperature conditions is necessary. *Geobacillus* are generally used in complex environments, and the number of genes coding for transposons implies the adaptability of *Geobacillus* to the environment (Frost et al., 2005). For example, the genome of *Geobacillus* sp. WCH70 has 125 annotation transpositions, which indicates that *Geobacillus* sp. WCH70 has a highly variable chromosomal, which can add or delete non-essential genes and gene clusters according to environmental conditions (Brumm et al., 2016).

Furthermore, many glycolytic thermophiles can use polymeric or short oligomeric carbohydrates with low nutritional requirements to produce lactic acid, formic acid, acetic acid, and ethanol as products (Niehaus et al., 1999; Taylor et al., 2009). Strains such as *Geobacillus thermoglucosidasius* DSM2542 have been developed for industrial bioethanol production from lignocellulosic feedstocks (Cripps et al., 2009; Chen et al., 2015). *Geobacillus* sp. Strain DUSELR13 has been developed for thermostable xylanase and ethanol production with lignocellulosic biomass (Bibra et al., 2018). *Geobacillus* sp. strain WSUCF1 is a thermophilic exopolysaccharide-producing bacterium and producing highly thermostable xylanase utilizing lignocellulosic biomas (Bhalla et al., 2014; Wang et al., 2019, 2021). Therefore, the study of *Geobacillus* as a significant source of thermostable enzymes and a platform host for lignocellulosic biomass natural products is critical (Chen et al., 2015).

*Geobacillus* sp. CX412 was isolated from Tengchong City, Yunnan Province, China. The genome of *Geobacillus* sp. CX412 strain was sequenced, and its metabolic potential was analyzed.

## Materials and methods

### Organism information

#### Classification and features

*Geobacillus* sp. CX412 is a novel thermophilic species obtained from hot spring soil in Tengchong City, Yunnan Province, China (24.953861° latitude and 98.443661° longitude). The organism was isolated from hot spring soil by enrichment and plating on a screening medium (screening medium contains (per liter) 8.0 g tryptone, 7.0 g casein, 3.0 g glucose, 5.0 g sodium chloride, 2.0 g disodium hydrogen phosphate, 10.0 g dehydrated calf brain extract, 15.0 g agar, pH 7.0–7.4) at 75°C.

### Genome sequencing information

Illumina Hiseq is used for sequencing to obtain the original data of the sequencing. FastQC assesses the quality of the original sequencing data, and then the Illumina sequencing data is cut by Trimmomatic (Bolger et al., 2014) to obtain relatively accurate and practical data. The Pacific Biosciences (PacBio) RS II is used for sequencing, and the original data is quality-cut to obtain high-quality data. Pacbio/single-molecule sequencing data were assembled using Canu (Koren et al., 2017), Illumina Hiseq sequencing data were introduced, and GapFiller (Boetzer and Pirovano, 2012) was used to complement the assembled scaffolds with GAP. Finally, sequence correction was performed using PrInSeS-G (Massouras et al., 2010). The editing errors and indels were fixed in segments during splicing. After obtaining the genome sequence, Prokka (Seemann, 2014) was used to predict the genetic elements: gene, tRNA, rRNA, etc. Sequencing was done at Sangon Biotech (Shanghai) Co., Ltd.

### Taxonomic assignment and phylogenetic analysis

The predicted 16S rRNA sequence was compared with the NCBI 16S database using NCBI Blast+ (Altschul et al., 1997) to obtain information on its homologous strains, and a phylogenetic tree was constructed. Download genome sequences of approximate strains, and perform average nucleotide identity (ANI) and digital DNA-DNA hybridization (DDH) were analyzed by JSpeciesWS and GGDC 3.0, respectively (Richter et al., 2016; Meier-Kolthoff et al., 2022).

### Functional annotation

NCBI Blast+ (Altschul et al., 1997) was used to compare the gene protein sequence with the COG database (Tatusov et al., 2000) to obtain its functional annotation information, KAAS (Kanehisa and Goto, 2000; Moriya et al., 2007) was used to obtain the gene KEGG annotation information, and HMMER3 (Eddy, 2009) was used to compare the gene protein sequence with the Carbohydrate active enzymes (CAZymes) database (Lombard et al., 2014) to obtain its functional annotation information.

### Accession numbers

The complete genome information of *Geobacillus* sp. CX412 was deposited in GenBank under the accession number CP103461-CP103464.

## Results and discussion

### Complete genome sequence of *Geobacillus* sp. CX412

**FIGURE 1:**
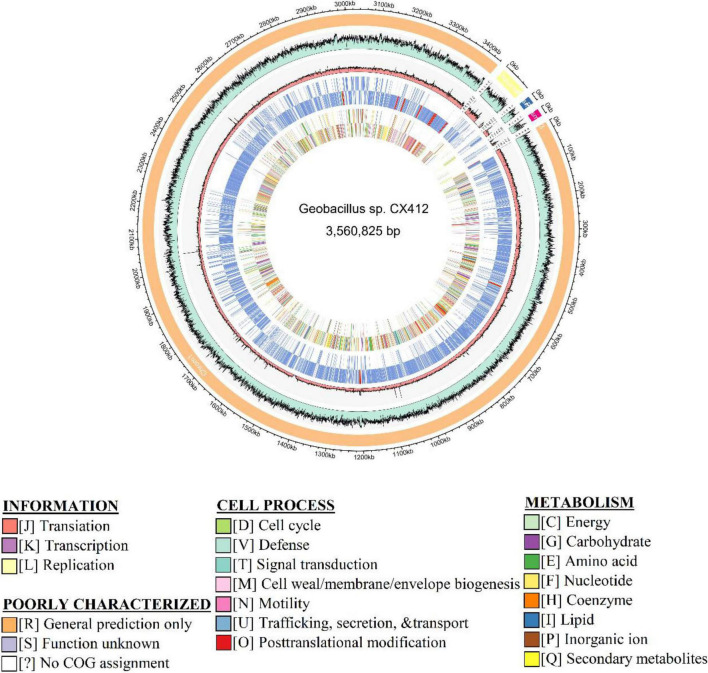
Genome map of *Geobacillus* sp. CX412. From outer to inner: Scale marks in kb, GC%, Coverage, Gene category, and COG category, respectively.

*Geobacillus* sp. CX412 is a Gram-positive, rod-shaped bacterium with an optimum growth temperature of 75°C and a maximum growth temperature of 85°C (Table 1). The total genome length of Geobacillus sp. CX412 was 3,560,825 bp, the average G + C content was 42.5%, and there were 91 tRNA genes and 26 rRNA genes (Table 2 and Figure 1). There are 3,763 predicted protein-coding regions in the genome (Table 2). A total of 2,678 genes (71.17%) were annotated in the COG database, and about 30% of the annotated genes were not assigned to COG or had unknown functions (Table 3).

**TABLE 1:** Classification and general features of *Geobacillus* sp. CX412.

Property	Term	Evidence code^a^
Classification	Domain *Bacteria*	TAS
	Phylum *Firmicutes*	TAS
	Class *Bacilli*	TAS
	Order *Bacillales*	TAS
	Family *Bacillaceae*	TAS
	Genus *Geobacillus*	TAS
	Species *Geobacillus* sp.	
	Strain: CX412	
Gram stain	Positive	IDE
Cell shape	Rods and chains of rods	IDE
Motility	Motile	IDE
Temperature	55–85°C	IDE
Optimum temperature	75°C	IDE
pH range; Optimum	5.8–8.0; 7.2	IDE
Carbon source	Carbohydrate or protein	IDE
Habitat	Thermal spring soil	IDE
Salinity	Not reported	IDE
Oxygen requirement	Aerobic	IDE
Biotic relationship	Free-living	IDE
Pathogenicity	Non-pathogen	IDE

^a^Evidence codes–IDE, Inferred from Direct Experiment; TAS, Traceable Author Statement (i.e., a direct report exists in the literature).

**TABLE 2:** Genome statistics of representative thermophilic *Geobacillus* and *Parageobacillus*.

Strain	*G.* sp. CX412	*G.* sp. WCH70	*P.* toebii NBRC 107807	*P.* thermoglucosidasius NBRC 107763	*P.* thermoglucosidasius C56-YS93
Origin	Yunnan, China	Middleton, WI, USA	Tokyo, Japan	Tokyo, Japan	USA
Genome size (bp)	3,560,825	3,508,804	3,263,973	3,871,162	3,993,793
G + C content (%)	42.45	42.80	42.15	43.69	43.93
Number of tRNA genes	91	92	89	81	90
Number of protein-coding genes	3,763	3,477	3,220	3,725	3,787

**TABLE 3:** Number of genes associated with general COG functional categories.

Code	Value	Percent	Description
J	169	4.49	Translation, ribosomal structure, and biogenesis
A	0	0.00	RNA processing and modification
K	158	4.20	Transcription
L	195	5.18	Replication, recombination, and repair
B	0	0.00	
D	40	1.06	Cell cycle control, cell division, chromosome partitioning
V	31	0.82	Defense mechanisms
T	112	2.98	Signal transduction mechanisms
M	116	3.08	Cell wall/membrane/envelope biogenesis
N	23	0.61	Cell motility
U	54	1.44	Intracellular trafficking, secretion, and vesicular transport
O	102	2.71	Posttranslational modification, protein turnover, chaperones
C	182	4.84	Energy production and conversion
G	149	3.96	Carbohydrate transport and metabolism
E	239	6.35	Amino acid transport and metabolism
F	75	1.99	Nucleotide transport and metabolism
H	136	3.61	Coenzyme transport and metabolism
I	77	2.05	Lipid transport and metabolism
P	160	4.25	Inorganic ion transport and metabolism
Q	44	1.17	Secondary metabolites biosynthesis, transport, and catabolism
R	343	9.12	General function prediction only
S	273	7.25	Function unknown
-	1,085	28.83	Not in COGs

### Taxonomic assignment and phylogenetic analysis

**FIGURE 2:**
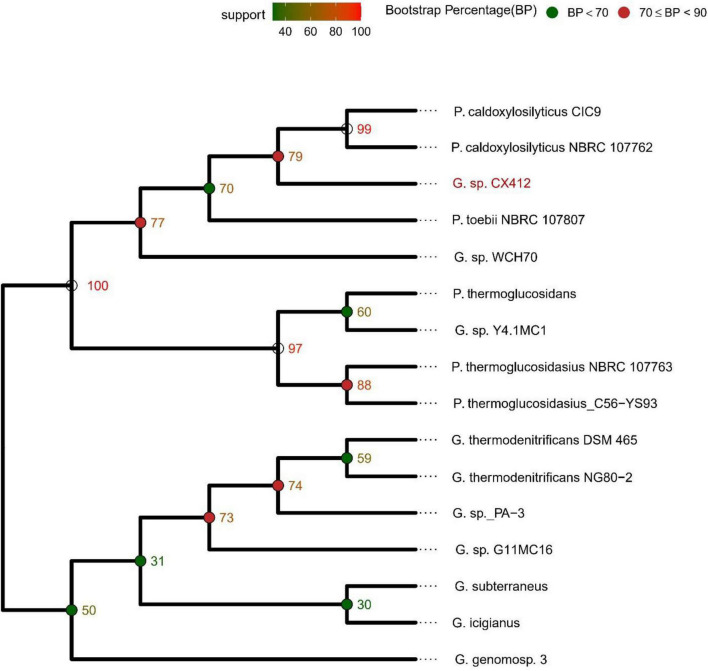
16S rRNA-based phylogenetic tree. G., *Geobacillus*; P., *Parageobacillus*.

After the 16S rRNA sequences were compared in the NCBI database, 16S rRNA sequences of the strains were selected according to the similarity to construct a phylogenetic tree. As shown in Figure 2, *Geobacillus* sp. CX412 is closely related to other *Geobacillus* and is an independent branch in the phylogenetic tree, confirming that it is *Geobacillus*. The four closest strains with complete genome sequences (Table 2) were selected for comparative analysis, and the results showed that these genomes shared 1,315 homologous gene clusters.

**FIGURE 3:**
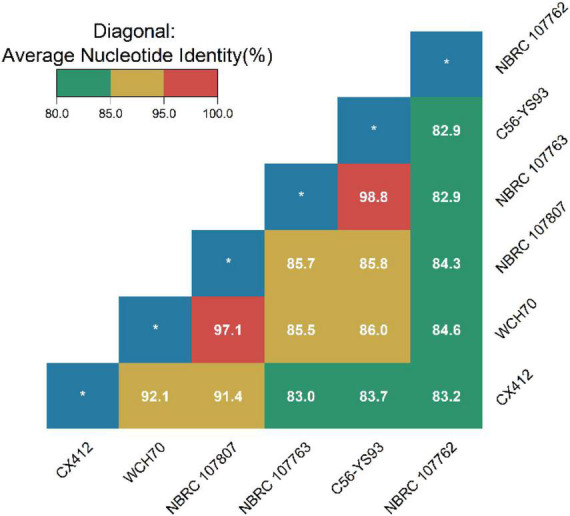
The average nucleotide identity (ANI) values (%). CX412, *Geobacillus* sp. CX412; WCH70, *Geobacillus* sp. WCH70; NBRC 107807, *Parageobacillus toebii* NBRC 107807; NBRC 107763, *Parageobacillus thermoglucosidasius* NBRC 107763; C56-YS93, *Parageobacillus thermoglucosidasius* C56-YS93; NBRC 107762, *Parageobacillus caldoxylosilyticus* NBRC 107762. *Represents self-ANI analysis with a theory of 100.

Average Nucleotide Identity (ANI) is an indicator for comparing the relatedness of two genomes at the nucleotide level. ANI is the average base similarity between homologous segments of two microbial genomes, characterized by a high degree of discrimination between closely related species. Compared with the traditional DDH, the calculation of the ANI index is simple and time-saving, and it is helpful to build a structured database, which is convenient for the follow-up research of bioinformatics scholars (Brumm et al., 2016). The ANI of *Geobacillus* sp. CX412 and the closely related strain *Geobacillus* sp. WCH70 was 92.1%, and the ANI of the strain *Parageobacillus toebii* NBRC 107807 was 91.4%, lower than the new species’ critical value of 95% (Figure 3). At the same time, the DDH of *Geobacillus* sp. CX412 and *Geobacillus* sp. WCH70 was 36.8%, and the DDH of *Parageobacillus toebii* 107,807 was 35.7%, lower than the new species’ critical value of 70% (Supplementary Table 1). This suggests that *Geobacillus* sp. CX412 should be a new *Geobacillus* sp.

### Comparison with other *Geobacillus*

In order to better understand the characteristics of *Geobacillus* sp. CX412, the number and metabolic potential of *Geobacillus* sp. CX412 and the other four species with complete genome sequence similarity were analyzed based on COG and CAZymes.

It shows that superoxide dismutase (SOD) is an essential protein for cells to resist high temperature, and Cu/Zn superoxide dismutase (SOD1) enzymes under Cu^2+^/Zn^2+^ presence can also make cells have high-temperature resistance (Askwith et al., 1994). At the same time, ClpP protein is found to affect the temperature resistance of the strain (Gerth et al., 2008). As a thermophilic bacterium, *Geobacillus* sp. strain CX412 should have genes related to high-temperature tolerance. The results showed that *Geobacillus* sp. CX412 had related genes for SOD, SOD1, and ClpP proteins; the related genes generally existed in the near-derived strains (Supplementary Table 2). The existence of related genes suggests the reasons for the high-temperature resistance of *Geobacillus* sp. CX412.

The ability of *Geobacillus* to thrive in this diverse and often harsh environment may be due to the predicted encoding transposons of many *Geobacillus* species (Bouzas et al., 2006; Bergquist et al., 2014). To some extent, the number of predicted coding transposons indicates the variability of the organism’s chromosomes, which can add or delete non-essential genes and gene clusters according to environmental conditions, representing the ability of the organism to adapt to the environment (Brumm et al., 2016). As shown in Table 4, *Geobacillus* sp. CX412 contained 73 predicted coding transposons. After comparing with the near-derived strains and reviewing the literature (Supplementary Table 3; Brumm et al., 2016), it was found that the predicted number of transposons encoded by *Geobacillus* sp. CX412 in *Geobacillus* was more than three times that of *Parageobacillus toebii* NBRC 107807. At the same time, the predicted number of transposons encoded by *Geobacillus* sp. CX412 in *Geobacillus* was significantly more than that of *Parageobacillus thermoglucosidasius* NBRC 107763 and *Parageobacillus thermoglucosidasius* C56-YS93. It shows that *Geobacillus* sp. CX412 also has a strong ability to adapt to the environment.

**TABLE 4:** Comparison of predicted transposons.

Function name	COG id	CX412	WCH70	107807	107763	C56-YS93
Transposase, IS605 family	COG0675	23	37	3	1	1
REP element-mobilizing transposase RayT	COG1943	3	5	0	1	0
Transposase InsO and inactivated derivatives	COG2801	3	3	1	5	10
Transposase InsE and inactivated derivatives	COG2963	1	0	0	1	6
Transposase, mutator type	COG3328	21	16	7	10	9
Transposase	COG3335	1	5	1	3	2
IS4 transposase InsG	COG3385	1	0	0	0	0
Transposase, IS66 family	COG3436	1	0	0	0	0
Transposase, IS204 family	COG3464	5	9	9	4	1
Transposase, IS116 family	COG3547	6	1	0	1	0
Transposase, IS1182 family	COG3666	3	1	1	2	4
Transposase	COG4584	1	0	0	4	16
Transposase	COG5421	4	7	0	0	0
Transposase	Not in CX412	0	4	1	3	1
Total		73	88	23	35	50

CX412, *Geobacillus* sp. CX412; WCH70, *Geobacillus* sp. WCH70; 107807, *Parageobacillus toebii* NBRC 107807; 107763, *Parageobacillus thermoglucosidasius* NBRC 107763; C56-YS93, *Parageobacillus thermoglucosidasius* C56-YS93.

KEGG (Kyoto Encyclopedia of Genes and Genomes) is a comprehensive database of biological systems that integrates genomic, chemical, and system functional information. KEGG GENES collects all known complete genome gene protein sequences, including the minimum information for each gene. The KO (KEGG ORTHOLOG) system links the various KEGG annotation systems together. After the KO annotation of the gene, the KEGG metabolic pathway classification is carried out according to the connection between the KO and pathway. There are seven categories: cellular processes, environmental information processing, genetic information processing, human diseases, metabolism, organismal systems, and drug development.

**FIGURE 4:**
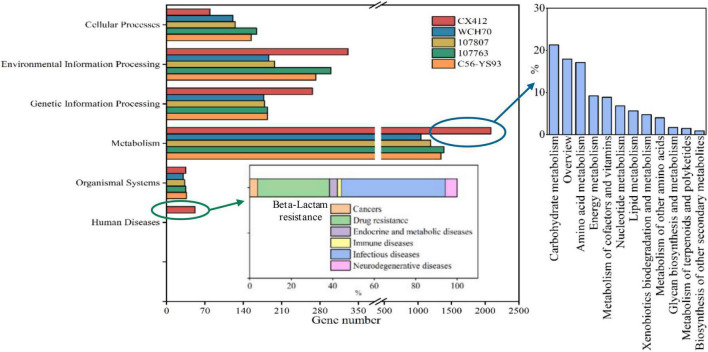
KEGG Pathway categories histogram. CX412, *Geobacillus* sp. CX412; WCH70, *Geobacillus* sp. WCH70; 107807, *Parageobacillus toebii* NBRC 107807; 107763, *Parageobacillus thermoglucosidasius* NBRC 107763; C56-YS93, *Parageobacillus thermoglucosidasius* C56-YS93.

In order to analyze the metabolic pathway of *Geobacillus* sp. CX412, the genes of *Geobacillus* sp. CX412 and other four near-derived strains were compared with the KEGG functional pathway database for functional annotation (Figure 4). The proportion of six functional genes of *Geobacillus* sp. CX412 was 2.8% respectively (cellular processes), 11.6% (environmental information processing), 9.3% (genetic information processing), 1.8% (human diseases), 73.2% (metabolism), 1.2% (organismal systems). It was indicated that there are six categories of functional genes of *Geobacillus* sp. CX412 (excluding drug development). At the same time, it can be seen from Figure 4 that the metabolic function genes of *Geobacillus* sp. CX412 are mainly carbohydrate metabolism and amino acid metabolism, and the metabolic function genes of *Geobacillus* sp. CX412 are significantly more than those of other near-derived strains (more than 50.5–100.1%). It was revealed that *Geobacillus* sp. CX412 is the strain with more robust metabolism in the genus *Geobacillus*. In addition, *Geobacillus* sp. CX412 has the human disease group that other near-derived strains do not have, in which the number of genes annotated to the ko00312 pathway (β-lactam resistance) accounts for 34.6% of the total genes associated with human disease. The near-derived strain *Geobacillus* sp. WCH70 of *Geobacillus* sp. CX412 was isolated from the aerobic fermenter (Brumm et al., 2016). Combined with the results of the KEGG analysis, it could be seen that *Geobacillus* sp. CX412 could also be used in composting, which had related genes of β-lactam resistance and more active metabolism (Figure 4). It also implies that the *Geobacillus* sp. CX412 has a broader range of applications.

**FIGURE 5:**
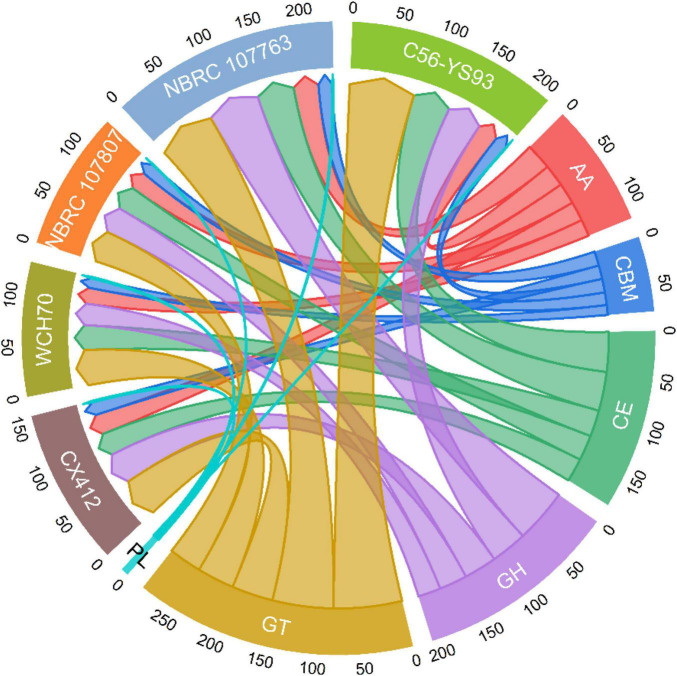
Carbohydrate-Active Enzymes (CAZymes). CAZymes classification result: AA, Auxiliary Activities; CBM, Carbohydrate-Binding Modules; CE, Carbohydrate Esterases; GH, Glycoside Hydrolases; GT, Glycosyl Transferases; PL, Polysaccharide Lyase; CX412, *Geobacillus* sp. CX412; WCH70, *Geobacillus* sp. WCH70; 107807, *Parageobacillus toebii* NBRC 107807; 107763, *Parageobacillus thermoglucosidasius* NBRC 107763; C56-YS93, *Parageobacillus thermoglucosidasius* C56-YS93.

Studies have also shown that *Geobacillus* species can grow in high-temperature environments (up to 70°C or more), and the advantages of using thermophilic bacteria as whole-cell biocatalysts include reducing the risk of contamination and accelerating biochemical processes in fermentation (Chen et al., 2015). Unexpectedly, *Geobacillus* sp. WCH70 lacks the predicted polysaccharide degradation clusters in many *Geobacillus* species, including metabolic clusters for hemicellulose degradation (Markowitz et al., 2014; Brumm et al., 2016). Nearly related strains of *Geobacillus* sp. CX412 include *Geobacillus* sp. WCH70. Therefore, to determine the metabolic potential of *Geobacillus* sp. CX412, CAZymes analysis was performed (Supplementary Table 4; Figure 5). CAZymes are divided into different families such as glycoside hydrolases (GH), glycosyltransferases (GT), carbohydrate-binding modules (CBM), carbohydrate esterases (CE), accessory activity (AA), and polysaccharide lyase (PL) (Lemos et al., 2017). *Geobacillus* sp. CX412 encompassing all six CAZymes families, as follows: 24.5% GHs, 30.8% GTs, 17.6% CEs, 15.7% AAs, 10.1% CBMs, and 1.3% PLs.

**FIGURE 6:**
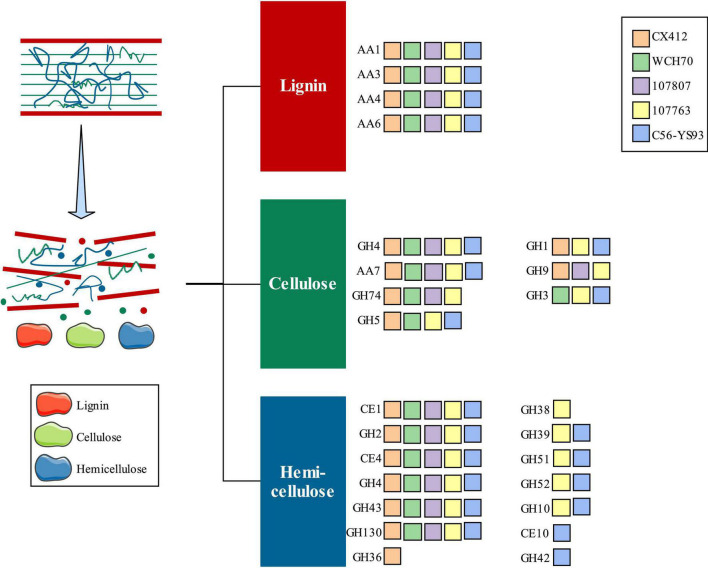
An overview of lignocellulose degradation. AA, Auxiliary Activities; CE, Carbohydrate Esterases; GH, Glycoside Hydrolases. CX412, *Geobacillus* sp. CX412; WCH70, *Geobacillus* sp. WCH70; 107807, *Parageobacillus toebii* NBRC 107807; 107763, *Parageobacillus thermoglucosidasius* NBRC 107763; C56-YS93, *Parageobacillus thermoglucosidasius* C56-YS93.

CAZymes are involved in constructing and breaking down complex carbohydrates and glycoconjugates in various biological processes (Lemos et al., 2017; Gavande et al., 2021). CBMs are the necessary modules for cellulolytic enzymes to bind to their substrates. AAs are involved in the degradation of lignin polymers, and CEs are the key to efficient hemicellulase activity. Cellulases and hemicellulases in GHs play an essential role in cellulose depolymerization (Gavande et al., 2021). Therefore, the genes encoding lignocellulose-degrading enzymes were screened, and 45 related genes were found in *Geobacillus* sp. CX412. Among them, there are 12 kinds of enzymes related to cellulolysis (GH1, GH4, GH5, GH9, GH74, and AA7) and 16 kinds of enzymes related to hemicellulose (GH2, GH4, GH36, GH43, GH130, CE1, and CE4), and 17 lignin oxidases (AA1, AA3, AA4, and AA6). The GH36 family is found only in the *Geobacillus* sp. CX412 genome (Figure 6). The GH36 family includes a thermostable hemicellulose (Lemos et al., 2017). *Parageobacillus thermoglucosidasius* C56-YS93, isolated from Yellowstone National Park in the United States, is a biomass degrader which can effectively degrade lignocellulose (Brumm et al., 2015c). *Geobacillus* sp. CX412 contains 18 lignocellulose-degrading enzymes, and *Parageobacillus thermoglucosidasius* C56-YS93 contains 22 kinds (Figure 6). Compared with *Parageobacillus thermoglucosidasius* C56-YS93, it can be found that *Geobacillus* sp. CX412 can also effectively degrade lignocellulose.

## Conclusion

*Geobacillus* sp. CX412 is a gram-positive, rod-shaped bacterium with an optimum growth temperature of 75°C, a maximum growth temperature of 85°C, and an average G + C content of 42.5%. There are 91 tRNA genes and 26 rRNA genes. Seventy-three predicted coding transposons indicate that *Geobacillus* sp. CX412 has a highly variable chromosome and that *Geobacillus* sp. CX412 has a strong ability to adapt to the environment. Compared with the near-derived strains with KEGG analysis, it was found that *Geobacillus* sp. CX412 has the unique β-lactam resistance and more active metabolism (more than 50.5–100.1%). It was also implied that the *Geobacillus* sp. CX412 has a broader range of applications. Analysis of the metabolic potential of *Geobacillus* sp. CX412 showed that *Geobacillus* sp. CX412 contained 45 genes related to lignocellulose degradation. Among them, there are 12 enzymes related to cellulolysis, 16 kinds of enzymes related to hemicellulose, and 17 lignin oxidases. *Geobacillus* sp. CX412 has the potential to efficiently degrade lignocellulose. These findings add to the growing library of known lignocellulose degradants and support further research into their biotechnological potential.

## Data availability statement

The data presented in this study are deposited in the GenBank repository, accession numbers: CP103461–CP103464.

## Author contributions

XL: methodology, software, data curation, writing – original draft, formal analysis, and validation. WZ: conceptualization, methodology, and validation. X-RZ: investigation and data curation. H-XH: investigation and validation. BD: conceptualization, resources, writing – review and editing, and supervision. All authors contributed to the article and approved the submitted version.

## Abbreviations

ANI: average nucleotide identity
DDH: digital DNA-DNA hybridization
ME: minimum-evolution
COG: cluster of orthologous groups of proteins
KEGG: Kyoto encyclopedia of genes and genomes
CAZymes: carbohydrate active enzymes.
