# Supplementary material for: Genome sequencing analysis of a novel thermophilic strain Geobacillus sp. CX412
Source: Front Microbiol. 2022 Nov 11;13:1035311. doi: 10.3389/fmicb.2022.1035311 (PMC9693769; doi:10.3389/fmicb.2022.1035311)
Supplement: Supplementary file 1 [file Data_Sheet_1.pdf]

Genome sequencing analysis of a novel thermophilic strain *Geobacillus* sp. CX412.

Xin Li[1], Wei Zhang[1,2], Xin-Ru Zhong[1], Hao-Xuan Han[1], Bin Dong[1,3*]

[1]School of Environmental Science and Engineering. Tongji University, Shanghai 200 092, PR China

[2]School of Environment and Architecture. University of Shanghai for Science and Technology, Shanghai 200093, PR China

[3]YANGTZE Eco-Environment Engineering Research Center, China Three Gorges Corporation, Beijing 100038, PR China

[*]Corresponding author:

Bin Dong

E-mail addresses: dongbin@tongji.edu.cn

Tel.: +86-13918126169.

Table S1 Digital DNA-DNA hybridization with *Geobacillus* sp. CX412

Query genome	Reference genome	DDH	Model C.I.	Distance	Prob. DDH >= 70%
CX412	WCH70	36.8	[33.4 - 40.3%]	0.4265	0.74
CX412	NBRC 107807	35.7	[32.3 - 39.2%]	0.4391	0.54

CX412: *Geobacillus* sp. CX412; WCH70: *Geobacillus* sp. WCH70; NBRC 107807: *Parageobacillus toebii* NBRC 107807; DDH: digital DNA-DNA hybridization.

Table S2 Comparison of predicted transposons

Function Name	COG id	CX412	WCH70	107807	107763	C56-YS93
Cu/Zn superoxide dismutase	COG2032	1	1	1	1	1
Superoxide dismutase	COG0605	2	2	2	2	2
Periplasmic serine proteases (ClpP class)	COG0616	1	1	1	1	1

CX412: *Geobacillus* sp. CX412; WCH70: *Geobacillus* sp. WCH70; 107807: *Parageobacillus toebii* NBRC 107807; 107763: *Parageobacillus thermoglucosidasius* NBRC 107763; C56-YS93: *Parageobacillus thermoglucosidasius* C56-YS93.

Table S3 Comparison of predicted transposons

Predicted transposons	CX412[a]	WCH70[2]	NBRC 107807[a]	NBRC 107763[2]	C56-YS93[2]
Total	73	125	23	24	26

[a]Inferred from direct computational analysis. CX412: *Geobacillus* sp. CX412; WCH70: *Geobacillus* sp. WCH70; NBRC 107807: *Parageobacillus toebii* NBRC 107807; NBRC 107763: *Parageobacillus thermoglucosidasius* NBRC 107763; C56-YS93: *Parageobacillus thermoglucosidasius* C56-YS93.

Table S4 Number of CDSs mapped to enzymes in the CAZymes database

CAZymes Class	CX412	WCH70	107807	107763	C56-YS93
GH	39	27	33	64	43
GT	49	44	39	72	81
CE	28	31	30	46	47
AA	25	20	23	31	26
CBM	16	12	15	17	16
PL	2	2	1	2	1

CAZymes: Carbohydrate-Active Enzymes; CX412: *Geobacillus* sp. CX412; WCH70: *Geobacillus* sp. WCH70; 107807: *Parageobacillus toebii* NBRC 107807; 107763: *Parageobacillus thermoglucosidasius* NBRC 107763; C56-YS93: *Parageobacillus thermoglucosidasius* C56-YS93.
